# Correlation Analysis of China's Foreign Trade Structure and Industrial Structure Based on Correlation and Mutual Influence

**DOI:** 10.1155/2022/3570781

**Published:** 2022-06-20

**Authors:** Yang Zhou, Chaoyi Chen

**Affiliations:** ^1^School of International Studies, Renmin University of China, Beijing 100872, China; ^2^Engineering School, The University of Michigan, Ann Arbor 48109, MI, USA

## Abstract

In recent years, China's foreign trade has continued to develop and achieved remarkable results, and the economic development trend has been steady and positive overall. However, although there are new opportunities for the development of China's foreign trade, it also faces new challenges and dilemmas. Therefore, in the current international trade situation, it is of great significance to study the impact of China's foreign trade on industrial structure and find the key points for the development of endogenous economic dynamics on this basis. China's industrial structure has undergone a clear process of upgrading and is a major trading country for industrially manufactured goods. The results of the analysis of the subsectors show that the trend of structural upgrading within the industry is equally significant. At the same time, there is often a significant trend of synergistic changes in the percentage of processing trade and the percentage change of trade in high-tech products. Based on this, this paper analyzes the correlation between China's foreign trade structure and industrial structure based on the correlation of mutual influence. The evolutionary algorithm has the advantages of a strong ability to search for global optimal solutions and good robustness, which is applied as the core algorithm of this paper. The study in this paper examines the interaction between industrial structure and trade structure from the perspective of matching the association between industrial structure and trade structure on the basis of measuring the industrial structure and trade structure at the regional level. The experimental results demonstrate the validity of the model in this paper.

## 1. Introduction

The process of industrial structure optimization of a country is the process of promoting the industrial structure toward rationalization and heightening by using the internal and external factors of industrial structure evolution. The trade structure is one of the main external factors affecting the change in industrial structure. Generally speaking, the foreign trade structure of a country not only reflects the domestic industrial structure and resource endowment status but also is influenced by the demand of foreign markets [1]. How the evolution of the trade structure changes largely affects the direction and speed of the optimization of the country's industrial structure. If the trade structure continuously promotes the optimization of industrial structure, it can promote the sustainable development of the national economy, so the coordinated development of both has a significant impact on the economic development of a country.

**Figure 1:**
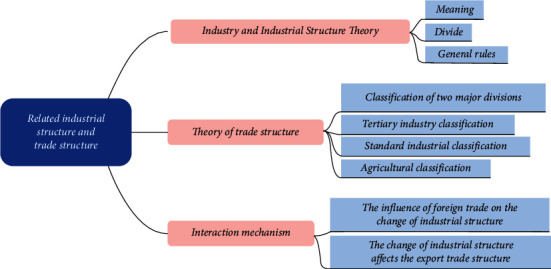
The division of industry and trade structure.

From the 1990s to the beginning of the 21st century, China's economy maintained high growth and opened up to the outside world, becoming a veritable “world factory.” But this high input, high consumption, and high pollution as the typical characteristics of the traditional industrial model cannot be sustained [2]. At the same time labor-intensive general trade, capital, and technology-intensive processing trade are the main trade mode that also appeared as a growth bottleneck. Nowadays, China is in the process of transforming from a “rough” economy to a “high-quality” economy, with economic growth continuing to slow down and the industrial structure in need of adjustment [3]. The report of the 19th Party Congress proposes accelerating the construction of an industrial system with synergistic development of real economy, science and technology innovation, modern finance, and human resources. However, China's exports of low-value-added products not only lack technical monopoly power and market competitiveness but also are vulnerable to control and blockage by developed countries in the process of climbing up the global value chain, facing the risk of “low-end lock” in the value chain, which in turn hinders the upgrading of industrial structure and economic. The division of industry and trade structure is shown in Figure 1.

At the same time, along with the weak global market, the sharp decline in import demand, the rise of international trade protectionism, and other situations, China's foreign trade environment is deteriorating [4]. Especially since 2018, the Sino-US trade dispute has again brought serious impact and instability to China's foreign trade industry. In the new international situation, China has to continuously adjust and optimize its industrial structure internally to achieve the general goal of supply-side structural reform and continuously strengthen its trade strength externally to realize the transformation from a large trading country to a strong trading country [5]. How to solve the overcapacity, optimize the trade structure, further adjust the industrial structure, and achieve sustainable economic development has become a major issue that needs to be addressed urgently. This paper will examine the interaction between industrial structure and trade structure, the regulation mechanism, and the evaluation criteria from the regional level [6]. In turn, it will identify possible differences among different regions in China and propose corresponding policy recommendations for their differences, making marginal contributions to enrich existing research from the perspective of research objects and research contents [7]. Based on this, this paper analyzes the correlation between China's foreign trade structure and industrial structure based on the correlation of mutual influence.

The main contributions of this paper are as follows: (1) China's industrial structure has undergone a significant upgrading process and is a major trading country for industrially manufactured goods. The paper first analyzes the results of the subindustry analysis showing that the trend of structural upgrading within industries is equally significant. Comparing the industrial structure and trade structure, there are different degrees of dislocation in the East, the Middle, and the West. At the same time, there is often a clear trend of synergistic changes in the proportion of processing trade and the proportion of trade in high-tech products. (2) This paper analyzes the correlation between China's foreign trade structure and industrial structure based on the correlation of mutual influence. Based on measuring the industrial structure and trade structure at the regional level, the research of this paper examines the interaction between industrial structure and trade structure from the perspective of correlation matching between industrial structure and trade structure. (3) Through relevant experiments, the results prove the correctness of the model of this paper.

## 2. Related Work

This section first presents the current state of research on foreign trade. Secondly, it introduces the current research status of the coevolutionary algorithm, which is widely used in the correlation analysis of trade structure and industrial structure. In this paper, foreign investment and trade refer to investment and trade from countries and regions other than China.

### 2.1. Research on Foreign Trade

**Figure 2:**
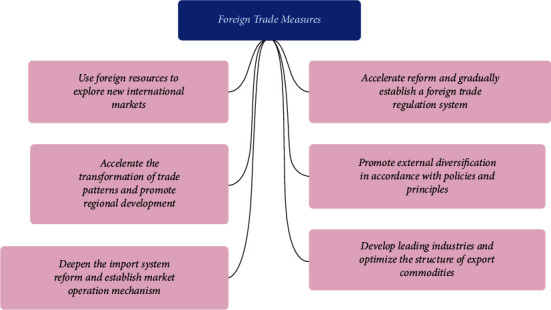
The effective measures for foreign trade.

The impact of foreign trade development on the industrial structure has significant research significance, and its research significance can be summarized into three aspects, namely, the significance of foreign trade development, the significance of industrial structure optimization, and the significance to enterprises engaged in international business [8]. First, from the point of view of the significance of foreign trade development, China's foreign trade is developing rapidly but is facing greater pressure at the same time. As the better and faster development of the national economy is the basis for better and faster development of foreign trade, the optimization and upgrading of industrial structure is the endogenous driving force for better and faster development of the national economy and foreign trade [9]. In terms of significance to the optimization and upgrading of industrial structure, the development of foreign trade will bring about not only an increase in the total amount of import and export and foreign direct investment but also the progress of technology and the accumulation of capital [10]. With the development of foreign trade, ecological environment, energy utilization efficiency, scientific and technological innovation ability, and human capital level will be affected to different degrees, all of which will have an impact on the adjustment of China's industrial structure. The effective measures for foreign trade are shown in Figure 2. Foreign trade measures mainly contain six indicators, which are (1) using foreign resources to develop new international markets, (2) accelerating the transformation of trade patterns and promoting regional development, (3) deepening the reform of the import system and establishing a market operation mechanism, (4) accelerating reform and gradually establishing a foreign trade supervision system, (5) promoting foreign diversification in accordance with policies and principles, and (6) developing leading industries and optimizing the structure of export commodities.

At present, foreign scholars have done more research on foreign trade and industrial structure but less on the impact of foreign trade development on industrial structure. On this basis, this paper will further comb the specific research on the impact of foreign trade development on industrial structure [11]. Due to the close relationship between the development of foreign trade, economic development, the development of industrial structure, and environmental changes, this paper also summarizes the relevant research on the relationship between foreign trade and economic and environmental impact [12]. Foreign scholars have empirically evaluated the overall welfare effect and structural adjustment of the Spanish economy after the trade liberalization of the European Economic Community and analyzed the relevant theories. This paper finds that the impact of increasing returns actually depends on the interaction between trade and domestic industrial policy [13]. Foreign scholars have done relatively little research on the impact of foreign trade development on industrial structure, while domestic scholars have done relatively more research in this regard. Most of these studies focus on the relationship between the development of foreign trade and the optimization and upgrading of industrial structure, and most of them only consider one aspect of the development of foreign trade or industrial structure. At the same time, they also lack targeted suggestions for enterprises [14]. This paper will improve this on the basis of previous studies. Through the analysis and collation of relevant literature and data, researchers and scholars conducted empirical research on a theoretical basis and studied the specific relationship between China's foreign trade, economic growth, and industrial structure upgrading [15].

Finally, it comes to the conclusion that the improvement of trade openness is conducive to the upgrading of industrial structure and puts forward many suggestions for the development of foreign trade and the optimization and upgrading of industrial structure [16]. Many scholars have conducted relevant research from the perspective of a foreign trade structure optimization, including relevant research on the interactive relationship between China's foreign trade structure and industrial structure, and creatively understood industrial structure optimization as industrial structure softening and industrial structure deepening, and put forward relevant constructive suggestions combined with empirical analysis [17]. Research scholars have conducted statistical measurement research on the optimization effect of China's foreign trade structure promoting industrial structure. Based on the complexity of international situation adjustment, this paper constructs the industrial structure optimization index under multiobjective measures of the types of foreign trade structures that affect the optimization effect of industrial structure and finally finds that there are four types of foreign trade structures that will have a significant impact on the industrial structure [18]. The article also summarizes the relevant conclusions that the structure of commodity technology level has not promoted the optimization of energy utilization level nor brought the improvement of benefits for many years and puts forward some suggestions on improving the technical level of industrially manufactured products, broadening the product types of industrially manufactured products, and promoting the formation of trade mode dominated by general trade [19].

By sorting out the relevant domestic and foreign research content, this paper finds that domestic research on the impact of foreign trade on the industrial structure is more abundant, while foreign scholars' research on this aspect is less, and most of the foreign scholars' research is focused on the relationship between foreign trade and economy and environment [20].

### 2.2. Research Status of Coevolution Algorithms

**Figure 3:**
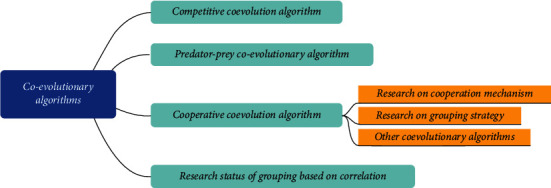
The current status of coevolutionary algorithm research.

This paper proposes a study on the correlation analysis of China's foreign trade structure and industrial structure using coevolutionary algorithms. The coevolutionary algorithm is an emerging class of algorithms proposed in recent years. It differs from the original evolutionary algorithms in that it takes into account the mutual coordination between populations and populations and between populations and environments in the evolutionary process based on the evolutionary algorithms. Since its introduction, the coevolutionary algorithm has achieved good results in both the solution of optimization problems and its application in many engineering fields [21, 22]. Coevolutionary algorithms are simulations of coevolutionary phenomena in ecology, and current research and experiments prove that coevolutionary strategies have become an effective way to improve the performance of evolutionary algorithms. The current status of coevolutionary algorithm research is shown in Figure 3.

The competitive coevolutionary algorithm refers to the relationship between two species that survive in the same area because they use the same resources, and if one of them occupies more resources, one of them must occupy fewer resources, and the relationship between the two populations is mutually damaging [23]. Coevolutionary algorithms divide an initial population into many subpopulations that evolve simultaneously, with both competitive and cooperative relationships between the subpopulations. In a competitive coevolutionary algorithm, the fitness of individuals is reflected by competition with individuals of other populations [24]. Scholars have mentioned in their studies that learning populations, competitive populations, and elite populations evolve simultaneously, and individuals in learning populations evolve by competing with individuals in competitive populations, and individuals who win the competition are rewarded, thus improving each other's ability to form an arms race phenomenon, and elite individuals obtained after each evolution are stored in elite populations [25]. Subsequently, scholars studied the problem of the adaptive grouping of decision variables in coevolutionary algorithms for research and proposed a coevolutionary algorithm model combining competition and cooperation, in which subpopulations compete for resources by means of competition, and then the winning side cooperates with each other to generate a solution after the competition is completed.

Some studies have also explored migration operations, interspecific dynamic adaptation, and diversity control of interspecific competitive coevolutionary algorithms. For example, based on the study of migration operations, an adaptive migration strategy is proposed, which specifically consists of adaptively adjusting the migration interval and size according to the evolutionary degree of the subpopulation itself [26]. In the coevolutionary algorithm, the predator-prey relationship essentially belongs to a competitive mechanism as well. In such a relationship, different species exist in both antagonistic and interdependent relationships [27]. So the predator-prey coevolutionary algorithm is a rough simulation of the model in the original ecological environment. Researchers use predation-prey relationships in the selection strategy of evolutionary algorithms to estimate the predation or predation ability of an individual based on its fitness value [28]. Not every individual is able to reproduce offspring, and when an individual wins a competitive predation relationship, that individual is able to continue reproducing its own offspring, i.e., that individual can get the opportunity to continue evolving [29]. The cooperative coevolutionary algorithm decomposes the optimization problem into many subproblems by simulating the cooperative symbiosis mechanism in ecology, and each subproblem will correspond to a subpopulation that evolves independently, and the subpopulations are cooperative with each other [30]. The most typical feature is that the individuals of each subpopulation represent only a part of the solution, while the solutions of many subpopulations are connected in the order of the original problem relevance to be the complete solution of the optimization problem.

## 3. Design of the Application Model

This paper proposes a study on the correlation analysis of China's foreign trade structure and industrial structure using a coevolutionary algorithm for the correlation interactions.  Section 3.1 introduces the underlying theory related to the algorithm, and Section 3.2 specifies the application of the algorithm in correlation interaction and correlation analysis.

### 3.1. Interdimensional Correlation Metric

Since its introduction, coevolutionary algorithms generally require decomposition of the problem when solving large-scale optimization problems, especially cooperative coevolutionary algorithms. When the dimensions of problems are interrelated, how to group them will not affect the relationship, so the correlation between dimensions becomes an important reason affecting the grouping. The correlation degree of test functions has always been the key factor that makes it difficult for evolutionary algorithms to solve complex optimization problems. If the correlation degree can be expressed by specific formulas, it will bring clear guidance for evolutionary algorithms to solve optimization problems. The relationship between loci in binary coding strings and the influence of different loci on the fitness of objective function can be expressed by the gene association model. At first, the gene association model is based on the pattern theorem and the building block hypothesis. In this theory, it is considered that the gene association model can be used to represent the difficulty of algorithm evolution. Firstly, the measurement of problem difficulty and gene correlation coefficient are defined, and their mathematical expressions are as follows:

(1) $$\begin{array}{c} \text{epiv}\left(f\right)=\frac{\sqrt{\sum_{x\in P}{\left(f\left(s\right)-\xi \left(s\right)\right)}^{2}}}{\sqrt{\sum_{t\in P}f^{2}\left(t\right)}},\\ \text{epic}\left(f\right)=\frac{\sum_{t\in P}\left(f\left(t\right)-{\bar{f}}_{P}\right)\left(\xi \left(t\right)-{\bar{\xi }}_{P}\right)}{\sqrt{\sum_{t\in P}{\left(f\left(t\right)-{\bar{f}}_{P}\right)}^{2}\sqrt{\sum_{t\in P}{\left(\xi \left(t\right)-{\bar{\xi }}_{P}\right)}^{2}}}}. \end{array}$$

The above metrics, gene association variance and gene association correlation coefficient, measure the difficulty of the problem, which is a measure at the overall level. The following metrics are based on the influence of gene positions on the fitness value of the function in information theory, and the correlation coefficient between gene positions and gene positions. In this approach, the interrelationship between problematic gene loci is tested by introducing the concepts of information entropy and mutual information quantity I. The information entropy is proposed to measure the uncertainty of the source, which is measured according to the probability of its occurrence. The mutual information quantity is also a valid information measure in information theory, which can reflect the correlation between two sets of events. The mutual information quantity can generally be calculated based on the difference between the information entropy and the conditional entropy.

**Figure 4:**
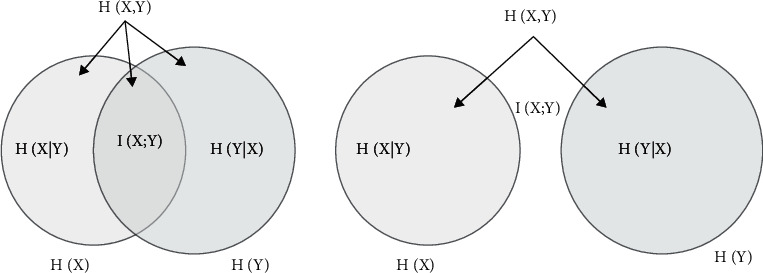
The relationship between information entropy and mutual information quantity when partially correlated.

Through some of the above formulas for finding a correlation, it is found that most of them link correlation with information entropy to solve for correlation based on information entropy. The amount of mutual information of the sample data itself cannot be calculated directly but needs to be indirectly reflected by the relationship between other values, so as to perform the correlation measure. The relationship between the information entropy and the amount of mutual information when partially correlated is shown in Figure 4.

Due to the paucity of test functions for evolutionary algorithms and the repeated use of many functions, the only functions that are available have a single fixed characteristic and can only be used for real-type solution function values; for the calculation of binary populations, a conversion process must exist. To this end, this paper designs a test function generator for enriching the variety of test problems that can be used directly for the calculation of binary populations without the need for precision conversion to real numbers. The construction method is inspired by the template theory and the NK model, and it is used as a theoretical basis to build a method for constructing test functions. According to the concept of template, it is possible to simply express the code of individuals with similar structures. With the concept of template, the process of population evolution in genetic algorithms can be approximated as a genetic operation through selection, crossover, and mutation and the process of continuously finding and replacing the template, so that the template will be the final optimal solution. The binary encoded function expressions are shown as follows:

(2) $$\begin{array}{c} a_{i}=\overset{4}{\underset{k=1}{\sum }}{\left(s_{k}⊕t_{k}\right)}_{i}. \end{array}$$

Subsequently, all submodules are summed to obtain the complete function value, as shown in the following mathematical expression.

(3) $$\begin{array}{c} \zeta_{ij}=\left\{\begin{array}{cc} 1-\frac{I\left(s_{i};f\left(x\right)\right)+I\left(s_{i};f\left(x\right)\right)}{I\left(s_{i},s_{j};f\left(x\right)\right)}, & I\left(s_{i},s_{j};f\left(x\right)\right)\neq 0,\\ 0, & \text{other.} \end{array}\right. \end{array}$$

The properties that can be flexibly controlled in this function set are homogeneity, correlation, continuity, and deception, respectively. The in-group correlation between dimensions within all submodules is higher than the out-group, and functions with this characteristic are well suited to be solved using the grouping strategy, and it is easy to find the optimal solution to the problem as long as the algorithm can find the grouping form in which the problem was designed before evolution. The new function is constructed to be directly invoked when in use, eliminating the need to convert binary to real numbers through precision and improving the disadvantage that the existing test function can only be used to compute real number codes.

### 3.2. Cooperative Coevolution Algorithm Based on Correlation Analysis

Coevolutionary algorithms have been successfully used to solve large-scale decomposable problems, and coevolution between subpopulations makes it easier to solve such problems. However, with the emergence of more and more incompletely divisible problems nowadays, the correlation between dimensions leads to the fact that the ordinary grouping form is no longer effective, and how to group becomes the crux of the problem. During the implementation of the new algorithm, in order to improve the performance of the algorithm and solve some problems encountered, three optimization operators are designed in this paper to assist in the implementation of the cooperative coevolutionary algorithm, namely, the sample design operator, the clustering operator, and the cooperative operator. In this paper, a sampling method based on the evolutionary process is designed. By designing samples, evolution and correlation measurement are closely related. Evolution direction can affect the correlation measurement, and the results of the measurement, in turn, affect the grouping strategy of the evolutionary process, thus improving the performance of the algorithm.

**Figure 5:**
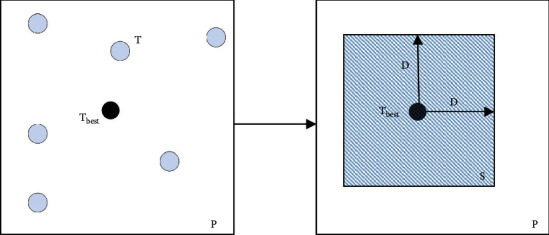
The generation process of region *S*.

This paper designs an operator to generate effective samples, which not only overcomes the problem of large sample size but also enables small samples to represent the correlation of the original population. Obtaining the optimal solution is the goal of evolutionary algorithms, but there are always some local optimal solutions near the optimal solution. In the early stage of evolution, a large number of locally optimal solutions will be produced, but with the evolution of iteration, local optimal solutions will gradually approach the global optimal. We set an offset *D* as the range of the generated region centered on the better individuals of the current evolution of the population and design the orthogonal operator conditional on this offset so that the samples in the small region are uniform enough. The generation process of region S is shown in Figure 5.

**Figure 6:**
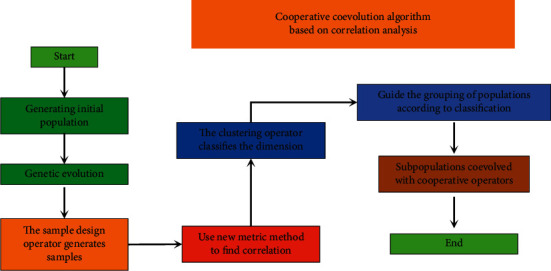
The algorithm flow chart.

The principle of designing orthogonal experiments in this paper is to select representative points where solutions are located from the experiment and to distribute them uniformly, which corresponds to an equal number of occurrences of different numbers in each column in the orthogonal table. The experimental design of this paper does not refer to the orthogonal table exactly but deals with individuals in a small area in an orthogonal manner under certain constraints, keeping the total size small while maintaining uniformity. The above sample design method not only improves the limitation of a large sample size of the original method but also makes sample uniformity through orthogonal experiment screening. At first, to select the current relatively optimal individual as the center to produce samples, not necessarily accurate measurement problems correlation, this is a process of iteration step by step; as the evolution process of iteration, the current optimal feasible solution can gradually be near global optimal solution, the correlation matrix measurement will become more accurate, in the form of the group, it also will be more and more accurate, and it is easier to find the optimal solution. The algorithm flow chart is shown in Figure 6.

Use sample design after each evolution operator is used to calculate the correlation matrix of the sample and the correlation matrix and then through the operator to find the grouping, clustering, and grouping as the upper encoding of the current population, and the upper coding within the process of the evolution of each species can at the same time also be updated, in the process of implementation in the upper coding and population in harmony as a whole. Population storage consists of two parts, namely, the current upper code and the real individual code. A sample is generated by designing the sample generation operator, and this sample is used to perform the correlation measure. After the correlation between problem dimensions is measured, the clustering operator is used to determine its appropriate grouping strategy, and the resulting grouping ordinal number is used as an upper-level code for the current evolutionary process to guide the population decomposition into subpopulations, which then evolve collaboratively with each other by cooperative complementarity using the cooperation operator.

## 4. Experiments and Results

This section uses the coevolutionary algorithm proposed in Section 3 for the validation of the correlation analysis between China's foreign trade structure and industrial structure. Firstly, it validates the algorithm of this paper for the correlation between China's total trade imports and exports and the trade balance. Secondly, the proposed algorithm is verified for the mutual impact between international investment and Chinese stock investment.

Experimental data were obtained from publicly available economic data from the Chinese government, including Chinese import and export investment from 2018 to 2020, and international direct investment in China from 2009 to 2018. The factors involved in the development of foreign trade will have a relevant impact on the industrial structure. The overall development is positive, and there are new opportunities while facing many challenges, which is an important feature of the current situation of China's foreign trade development. The software and hardware environment for this experiment is shown in Table 1.

**Figure 7:**
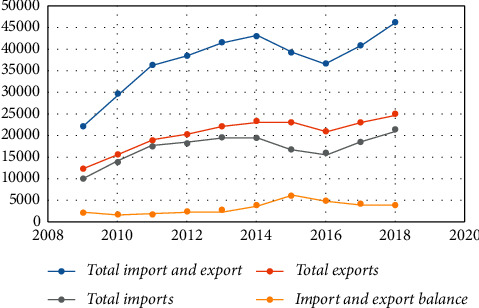
The current status of China's import and export of goods trade.

From the overall environment of international trade, although the current market demand is increasing, the trade barriers between countries have not weakened. Along with the negative impact of increased trade protectionism on global trade growth, Chinese products are facing greater problems and dilemmas in import and export. From the general trend of the development of the domestic economic environment, the continuous expansion of the total size of China's economy, the expansion of the scale of foreign investment, the faster growth of fixed asset investment, the accelerated upgrading of the economic structure, and the rise of comprehensive economic strength have all added endogenous impetus to the sustainable development of the national economy and laid the foundation for the development of China's import and export trade. The current status of China's import and export of goods trade is shown in Table 2 and Figure 7. In Figure 7, the horizontal coordinates indicate the year, and the vertical coordinates indicate the import and export trade volume.

**Figure 8:**
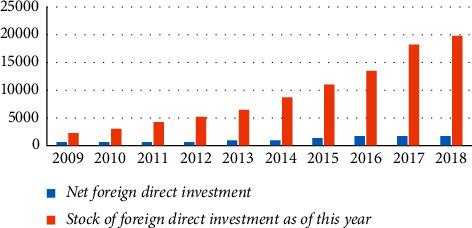
The current situation of China's net foreign direct investment and stock.

It can be seen that from 2009 to 2018, China's total exports, imports, and total imports and exports of goods trade showed an increasing trend, while the import and export surplus decreased in the past two years. Total exports of goods trade increased from the US $1201.61 billion in 2009 to the US $248668 billion in 2018, while total imports of goods trade increased from the US $1005.92 billion in 2009 to the US $2135.73 billion in 2018, both of which nearly doubled. Based on relevant data that can be found, although China's total imports and export calendar year continue to increase, the difference between service trade and merchandise trade is still large; although these all represent China's economic development and stability in the foreign trade development toward a good trend has not changed, still there are some structural problems. The overall stock of China's foreign direct investment showed an obvious increase trend, but China's net foreign direct investment showed an obvious decline trend from 2017 to 2018. The current situation of China's net foreign direct investment and stock is shown in Table 3 and Figure 8. In Figure 8, the horizontal coordinates indicate the year, and the vertical coordinates indicate the investment and stock market amounts.

At present, the main purpose of China's “going out” policy is to regulate the specific behavior of enterprises in OFDI, prevent the risks faced in the current international economic environment, improve the ability and level of OFDI, guide the main investment direction of enterprises in their main business and related businesses, control the quantity of investment, ensure the quality and height of investment, and guarantee the authenticity and reasonableness of investment. The Chinese government has even made a policy decision to control the quantity of investment, ensure the quality and height of investment, and guarantee the authenticity and reasonableness of investment. To this end, the Chinese government has even tightened its policies to make enterprises respond rationally to investments. The reason for the “inward” policy is that China is in the stage of industrial transformation and upgrading, and in order to promote a more rational industrial pattern in the country and to further advance the progress of industrial structure reform, it is necessary to further introduce advanced foreign technologies and management methods.

## 5. Conclusion

After combining the relevant theories and studying the overall situation of the changes in foreign trade and industrial structure over the years, this paper finds that in recent years, with the continuous rise of China's foreign trade level, the industrial structure also tends to be advanced and rationalized. However, in the case of high dependence on foreign trade, China is still facing greater foreign trade risks and pressures, while China's industrial structure also has a greater space for development. This paper measures the long-term impact of foreign trade development on industrial structure and the extent of short-term shocks, respectively. The paper finds that there is a long-run equilibrium relationship between China's foreign trade development level and different levels of industrial structure optimization indicators. Subsequently, after further analysis of the empirical results, this paper finds that, as the overall level of China's foreign trade continues to improve, the industrial structure is also being optimized, but in terms of the long-term equilibrium relationship, China's technological innovation capability may be somewhat inhibited.

Finally, on the basis of the above research, this paper also provides relevant ideas for the government and enterprises to promote industrial structure optimization and upgrading through foreign trade development, respectively, taking into account the efforts made by the Chinese government and relevant enterprises in the development of foreign trade and industrial structure optimization. The government, first of all, should promote the improvement of the overall quality of foreign trade by actively participating in the construction of the foreign trade environment and adjusting various aspects of domestic economic development. The country should hedge the risks of high foreign trade dependence by diversifying the export structure and increasing innovation investment and efforts, especially the risks of dependence on research and innovation. The government should strengthen trade relations with other countries and find more export and import substitutes for the country through international trade with more countries to change the situation of high regional concentration of imports and exports. In our future research plan, we plan to use recurrent neural networks and knowledge mapping to analyze the correlation study between China's foreign trade structure and industrial structure.

## Figures and Tables

**Table 1:** Experimental environment.

CPU	i5-9700 CPU @ 3.00 GHz
GPU	GTX TITAN X
Graphics memory	12G
Operating system	Ubuntu 16.04 LTS
CUDA	10.0
Deep learning framework	Pytorch

**Table 2:** The current status of China's import and export of goods trade.

Years	Total import and export (billion/$)	Total exports (billion/$)	Total imports (billion/$)	Import and export balance (billion/$)
2009	22075.3	12016.1	10059.2	1956.9
2010	29740.0	15777.5	13962.5	1815.0
2011	36418.6	18983.8	17434.8	1549.0
2012	38671.2	20487.1	18184.1	2303.0
2013	41589.9	22090.0	19499.9	2590.1
2014	43015.4	23423.0	19592.4	3830.6
2015	39530.3	22734.7	16795.6	5939.1
2016	36855.6	20976.3	15879.3	5097.0
2017	41071.4	22633.5	18437.9	4195.6
2018	46224.1	24866.8	21357.3	3509.5

**Table 3:** The current situation of China's net foreign direct investment and stock.

Years	Net foreign direct investment (billion/$)	The stock of foreign direct investment as of this year (billion/$)
2009	565.3	2457.6
2010	688.1	3172.1
2011	746.5	4247.8
2012	878.0	5319.4
2013	1078.4	6604.8
2014	1231.2	8826.4
2015	1456.7	10978.7
2016	1961.5	13573.9
2017	1582.9	18090.4
2018	1430.4	19822.7

## Data Availability

The dataset used in this paper is available from the corresponding author upon request.
